# Measurements of Tear Evaporation Rate in Subjects with Refractive Errors Using a Portable Evaporimeter

**DOI:** 10.3390/healthcare10020405

**Published:** 2022-02-21

**Authors:** Raied Fagehi, Gamal A. El-Hiti, Mushawwat H. Alsubaie, Ali Abusharha, Mana A. Alanazi, Ali M. Masmali, Turki Almubrad

**Affiliations:** Department of Optometry, College of Applied Medical Sciences, King Saud University, Riyadh 11433, Saudi Arabia; mesho66396@gmail.com (M.H.A.); aabusharha@ksu.edu.sa (A.A.); amana@ksu.edu.sa (M.A.A.); amasmali@ksu.edu.sa (A.M.M.); turkim@ksu.edu.sa (T.A.)

**Keywords:** refractive errors, dry eye, tear evaporation rate, ocular tear film stability, portable evaporimeter

## Abstract

Dry eye symptoms are associated with refractive errors. We aimed to measure the tear evaporation rate (TER) in subjects with refractive errors (RE) using a portable evaporimeter. This nonrandomized, case–control, and observational study included 75 subjects, including 25 subjects aged 18–38 years (28.8 ± 6.8 years) with myopia (−0.75 to −3.75 D) and 25 subjects aged 18–39 years (27.7 ± 5.5 years) with hyperopia (+0.50 to +3.75 D). In addition, a control group of 25 subjects with emmetropic eyes aged 20–30 years (23.6 ± 2.6 years) was recruited. The ocular surface disease index (OSDI) was completed, followed by the TER measurements using a portable evaporimeter. The OSDI score and TER measurements showed dry eye symptoms in 36% and 48% of myopic subjects, respectively. For hyperopic participants, dry eye was observed in 24% and 56% of the subjects based on the OSDI and TER scores, respectively. Significant differences (Mann-Whitney *U* test; *p* < 0.001) were found among the OSDI and TER scores recorded within the study (myopic and hyperopic subjects) and control groups. Significant strong positive correlations were detected between the OSDI and TER scores in both myopic (*p* = 0.004; *r* = 0.559) and hyperopic (*p* = 0.001; *r* = 0.619) subjects. The TER scores were significantly higher in subjects with RE (myopic and hyperopic) as compared with individuals with normal eyes.

## 1. Introduction

The mismatch between optical power and axial length leads to refractive errors (RE). RE cause blurriness and are considered one of the main causes of vision impairment [1]. In recent years, the prevalence of RE, and in particular myopia, has been increasing rapidly. It has been estimated that in 2010, uncorrected RE led to visual impairment (moderate to severe) in more than 100 million individuals [2]. The hyperopic eye is relatively underpowered, which results in objects at a near distance appearing blurry, while distant objects are seen clearly. In the hyperopic eye, the RE results from the short axial eye length compared to its optical power [1]. In contrast, the myopic eye has a long axial eye length compared with its power and is relatively overpowered. For myopic eyes, far objects appear blurry but less so at a short distance [1]. The prevalence of hyperopia and myopia varies between countries; there are environmental and genetic factors, and both conditions are found to be higher among adults (30.9% and 26.5%, respectively) than among children (4.6% and 11.7%, respectively) [3]. Hyperopia declines with increasing age, whereas myopia increases quickly as an individual ages [4,5]. The implications for the development of RE are highly dependent on the time spent using electronic devices. New technology offers the opportunity for RE correction. In addition, factors contributing to RE should be better understood and causative factors addressed. The association between dry eye and RE has also been investigated [6,7,8].

Dry eye is a common ocular disorder that affects a large proportion of the global population [9,10,11]. Dry eye causes a disruption in tear film stability, leading to various undesirable symptoms [11]. These symptoms vary from ocular discomfort to damage to the cornea and tear film [12]. Two major types of dry eye are known and are due to either excessive evaporation or deficiency of tears [13]. The excessive evaporation of tears increases osmolarity and is responsible for the thinning of the tear film [14,15]. The dysfunction of the meibomian gland is the major cause of dry eye that leads to hyperosmolarity within the tear film [16]. The lipid layer produced by the meibomian gland spreads over the tear film with each blink to reduce tear evaporation [17]. Therefore, the health of the meibomian gland is crucial for maintaining tear film stability and function. The structure of the tear film is complex; therefore, no single method can be used to detect all symptoms of dry eye [10]. Measurements of tear volume, production, evaporation, stability, and osmolarity are the most common methods for the detection of dry eye, along with the use of patient questionnaires [18,19,20,21].

The tear evaporation rate (TER) has been measured efficiently using a portable evaporimeter [22,23]. The use of a portable evaporimeter for measuring TER is quick, convenient, repeatable, and noninvasive as compared with other techniques [24]. A measurement of greater than 25 g/m^2^h at room temperature and a humidity of about 30% is a sign of dry eye [25]. In this study, we perform the first investigation of TER in subjects with RE (myopic and hyperopic) using a portable evaporimeter. We hypothesized that RE could have a negative effect on the tear film and act as a risk factor for dry eye.

## 2. Materials and Methods

### 2.1. Subjects

This nonrandomized, case–control, and observational study included 75 subjects. There were 25 subjects aged 18–38 years (28.8 ± 6.8 years; 9 women and 16 men) with myopia (−0.75 to −3.75 D) and 25 subjects aged 18–39 years (27.7 ± 5.5 years; 7 women and 18 men) with hyperopia (0.50 to 3.75 D). Both myopic and hyperopic participants’ eyesights were corrected with their spectacles. In addition, a control group of 25 subjects with normal eyes aged 20–30 years (23.6 ± 2.6 years; 12 women and 13 men) was recruited for comparison. The refractive errors for the subjects in the control group were assessed and were less than 0.5. Objective refraction was used to determine the RE of the subjects in a dim-light clinic. The subjects were recruited from Riyadh City, Saudi Arabia. We excluded subjects with a high cholesterol level (>4 mmol/L), a high body mass index (>24.9 kg/m^2^), hypertension, vitamin A and D deficiencies, thyroid disorders, anemia, and diabetes. In addition, smokers, pregnant and breast-feeding women, contact-lens wearers, and those with a history of ocular surgery and corneal diseases were excluded. Ethical approval was obtained, and written informed consent was provided by each participant before the start of the measurements. The research complied with the guidelines of the Declaration of Helsinki [26].

### 2.2. OSDI

The OSDI was completed by each participant first. A score of more than 13 was assigned as a dry eye [27].

### 2.3. TER Test

TER was measured using a portable evaporimeter obtained from Delfin Technologies (Surrey, UK). We performed the TER test three consecutive times for each participant. We allowed a gap of two minutes between the tests and calculated the average measurement. TER measurements were performed when both eyes were open, with normal blinking, and then closed. The TER test was performed in both eyes for each subject. The TER was calculated by subtracting the score obtained with both eyes closed from that recorded when both eyes were open. A dry eye was assigned for a measurement of more than 25 g/m^2^h. TER measurements were performed by the same examiner, and the environment was controlled in terms of humidity (<35%), temperature (22 °C), and airflow.

### 2.4. Statistical Analysis

Data were recorded using Microsoft Excel 2010 (Microsoft Corporation, Redmond, WA, USA) and analyzed using SPSS version 22 (IBM, Armonk, NY, USA). Spearman correlation coefficient was used to test the association between different scores [28]. The data were not normally distributed (Kolmogorov-Smirnov test; *p* < 0.05) for the OSDI and TER scores. The Mann-Whitney *U* test was used to study the correlation (Spearman’s rank correlation coefficient; *r*) between parameters. The median (interquartile range) was used to represent the average scores.

## 3. Results

The study included 25 subjects with myopia (−1.91 ± −0.87 D) and 25 hyperopic subjects (1.65 ± 0.82 D) alongside a control group (*N* = 25) with normal eyes. Table 1 shows the median OSDI and TER scores and the mean subject age among the study and control groups. The median TER score (27.0–28.3 g/m^2^h) in both myopic and hyperopic subjects showed mild symptoms of dry eye.

**Figure 1:**
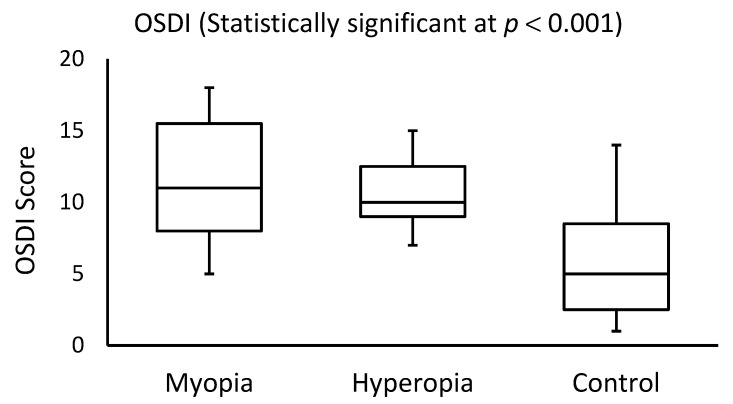
Side-by-side boxplots for the OSDI scores in myopia, hyperopia, and control groups.

**Figure 2:**
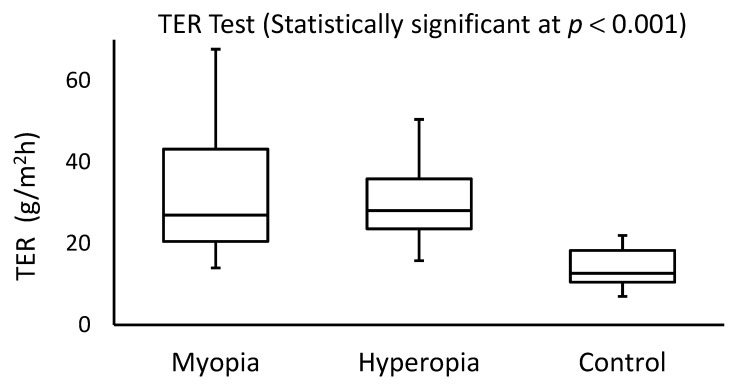
Side-by-side boxplots for the TER scores in the myopia, hyperopia, and control groups.

The OSDI score and TER measurements showed dry eye symptoms in 36% (*N* = 9) and 48% (*N* = 12) of myopic subjects, respectively. For hyperopic participants, dry eye was observed in 24% (*N* = 6) and 56% (*N* = 14) of the subjects based on the OSDI and TER scores, respectively. Significant differences (Mann-Whitney *U* test; *p* < 0.001) were detected among the OSDI and TER scores recorded within the study (myopic and hyperopic subjects) and control groups. No significant differences were found among the OSDI (Mann-Whitney *U* test; *p* = 0.830) or TER (*p* = 0.560) scores in either the myopic or hyperopic subjects. Figure 1 and Figure 2 display the side-by-side boxplots for the OSDI and TER scores for myopic, hyperopic, and control groups, respectively. Clearly, the OSDI and TER scores were significantly (*p* < 0.001) higher in myopic and hyperopic subjects as compared with those for the control group. The average coefficients of variance among the three TER scores in both myopic and hyperopic subjects were low (0.7% and 0.1%, respectively).

Significant strong positive correlations were found between the OSDI and TER scores in both myopic (*p* = 0.004; *r* = 0.559) and hyperopic (*p* = 0.001; *r* = 0.619) subjects. For the control group, a significant (*p* = 0.046) medium negative correlation (*r* = −0.402) was detected between the scores obtained from the OSDI and TER test. There were no significant (*p* > 0.05) differences among the scores collected from the OSDI and TER for myopic and hyperopic subjects.

## 4. Discussion

Individuals with dry eye often experience poor vision in the form of various symptoms that are difficult to assess using a single test [29]. Moreover, the correction between the scores from different tests to detect dry eye is often poor [30]. The volume or production of tears can be assessed using the Schirmer or phenol red thread (PRT) tests [19]. The tear film stability can be measured using the tear breakup time (TBUT) test [31], and the concentration of electrolytes in the tear film can be assessed using the osmolarity test [32]. The application of a TER test measures the rate of evaporation of water from the eye [33].

For RE subjects, the light cannot bend correctly because of the uneven shape of the eye, which leads to the lack of light concentration to focus on an image. Therefore, uncorrected RE has long-term implications on patient quality of life, particularly among children [34]. The current study suggests that subjects with RE (myopia and hyperopia) have a significantly high level of dry eye compared with participants with normal eyes. Both the median scores from the OSDI and TER measurements were higher than those recorded for subjects with normal eyes. No significant differences were found in the OSDI and TER scores between myopic and hyperopic subjects. Recently, a Delfin VapoMeter was used to assess the TER of chronic smokers and of subjects with thyroid gland disorder [22,23]. In smokers (*N* = 120; 25.4 ± 5.8 years), the median TER score was significantly (*p* < 0.05) higher (37.7 (59.3) g/m^2^h) compared with the control group (15.4 (13.1) g/m^2^h) [23]. The TER scores indicated that the majority of smokers (*N* = 85) have dry eye. Similarly, the TER was significantly (*p* < 0.05) higher in subjects with thyroid gland disorder (*N* = 20; 34.3 ± 6.3 years) compared with the control group (*N* = 20; 32.5 ± 5.1 years). The median TER was 41.2 (41.4) g/m^2^h and 15.7 (13.7) g/m^2^h in thyroid and normal eye subjects, respectively [22].

Previous studies have suggested the association between RE and dry eye, based on other methods for the detection of dry eye symptoms [6,7,8,35]. For example, the prevalence of dry eye symptoms among subjects with hyperopia (*N* = 45; 22.0 ± 2.4 years) was 26.6% based on Schirmer’s test, compared with only 1.1% for myopic subjects (*N* = 45; 22.6 ± 3.3 years) [6]. There was no significant difference (*p* = 0.413) between the mean tear volume among hyperopic (13.2 ± 5.0 mm) and myopic subjects (18.4 ± 4.3 mm) [6]. Subjects with hyperopia (*N* = 48) and myopia (*N* = 31) have a shorter TBUT (9.4 and 9.7 s, respectively) compared with normal eye subjects [7]. The prevalence of dry eye was found to be high among hyperopic (17.4%) and myopic subjects (36.5%) based on TBUT scores [7]. The correlations between the TBUT and hyperopic (*r* = −0.405) and myopic (*r* = 0.295) subjects were significant (*p* ≤ 0.05) [7]. However, measurements of tear production using the tear meniscus height test in hyperopic and myopic subjects showed no significant difference compared with measurements in subjects with normal eyes [7]. Dry eye symptoms (*p* = 0.004) and blurred vision (*p* = 0.003) were found to be high among subjects with uncorrected RE [8]. In addition, the prevalence of dry eye was high among relatively older subjects (10.8–12.0% for those aged 26–40 years) with RE compared with younger individuals (5.8–6.5% for those aged 16–25 years) [8]. Recently, the use of the tear ferning (TF) test has been used to establish the association between dry eye and RE [36]. In addition, the repeatability and reproducibility of the TER test using a VapoMeter have recently been approved [37].

Although we found a correlation between RE and dry eye, future studies including more participants and individuals with a high level of RE (e.g., moderate and severe) are still needed. In addition, the TER should be assessed among subjects with other types of RE (e.g., astigmatisms). Moreover, the mechanism by which RE induces a level of dry eye needs to be investigated.

## 5. Conclusions

Subjects with refractive errors have a significant level of dry eye. The OSDI and TER scores in myopic and hyperopic subjects were found to be significantly higher compared with those who have healthy normal eyes.

## Figures and Tables

**Table 1:** Average age, OSDI, and TER scores for subjects with myopia, hyperopia, and normal eyes (control group).

Parameter	Mean ± SD or Median (IQR)		
	Myopia (*N* = 25)	Hyperopia (*N* = 25)	Control (*N* = 25)
Age (year)	28.8 ± 6.8	27.7 ± 5.5	23.6 ± 2.6
OSDI	11.0 (7.5)	10.0 (3.5)	5.0 (6.0)
TER (g/m^2^h)	27.0 (22.7)	28.3 (11.8)	12.7 (7.8)

## Data Availability

Data are contained within the article.
